# Impact of Medicaid coverage expansion under the Affordable Care Act on mammography and pap tests utilization among low-income women

**DOI:** 10.1371/journal.pone.0214886

**Published:** 2019-04-03

**Authors:** Abeer G. Alharbi, M. Mahmud Khan, Ronnie Horner, Heather Brandt, Cole Chapman

**Affiliations:** 1 Health Services Policy and Management Department, Arnold School of Public Health, University of South Carolina, Columbia, South Carolina, United States of America; 2 Health Promotion, Education, and Behavior Department, Arnold School of Public Health, University of South Carolina, Columbia, South Carolina, United States of America; Centro per lo Studio e la Prevenzione Oncologica, ITALY

## Abstract

**Introduction:**

The Affordable Care Act (ACA) expanded the coverage of Medicaid to include entire population with income below 138% of federal poverty line. It remains unclear whether this policy change has improved access to and utilization of health care, particularly use of mammography and Pap tests among poor women.

**Methods:**

We used a difference-in-difference (DID) design to estimate the impact of Medicaid expansion on mammography and Pap tests utilization among low-income women. Expansion states are the treatment group and non-expansion states are the control group. The years 2012–13 are the pre-expansion period and 2015–16 are the post-expansion period for the purpose of estimating the DID parameters.

**Results:**

The difference-in-difference estimate show that likelihood of utilizing mammograms did not change significantly among low-income women after the implementation of Medicaid expansion (DID coefficient -0.0476 with t-statistics at -1.26), Pap test decreased (coefficient -0.0615, t-statistics -2.76), and Medicaid enrollment has increased significantly among low-income women living in expansion states (coefficient 0.0889 with t-value of 3.68).

**Conclusion:**

Expansion of Medicaid was associated with increased Medicaid enrollment but did not yield near-term improvement in use of mammography and Pap tests among low-income women. Factors beyond health insurance coverage may be important in determining the likelihood of obtaining these screenings. Policy makers should try to identify other barriers to cancer screenings among low-income women in the USA.

## Introduction

Breast cancer is the most commonly diagnosed cancer among American women, and the second most common cause of death from cancer besides lung cancer [1,2]. Cervical cancer incidence rates declined by half between 1975 and 2014 due to the widespread uptake of the Pap test, but declines have slowed down in recent years [1,2]. Evidence show that women who appropriately screen for breast and cervical cancer are likely to receive more timely diagnosis and treatment [3–10] and yet, rates of mammography and Pap test screenings remained suboptimal in the United States [11]. Low-income women utilize less screenings than middle or high income women. In 2015, 54.9% of low-income women received mammography while 60% received Pap test [11]. Goals of Healthy People 2020 include increasing the proportion of women who get mammograms to 81%, and Pap tests to 93%, based on the most recent guidelines [12]. There are several possible reasons for the suboptimal screening rates, among which lack of health insurance coverage is considered an important one. There is evidence that health insurance is associated with uptake of mammogram and Pap test use [13–17].

The Patient Protection and Affordable Care Act of 2010 (ACA) expanded the coverage of Medicaid to include the entire population aged 18–64 with income below 138% of the federal poverty line [18]. Since uninsured adults were more likely to be low-income, Medicaid expansion has the potential for improving access to health care among this poor segment of the population [19–21]. One important role of health insurance is reducing the cost of receiving preventive care and in turn, reduce costly events from poorly or unmanaged chronic conditions. Under ACA, participation of States in Medicaid expansion became optional after a supreme court ruling in 2012 [22] but many states decided to participate in Medicaid expansion immediately after the policy change and by September 2015, majority of the states have expanded Medicaid. As of September 2018, 34 states have adopted Medicaid expansion, 3 states are considering expanding, and 14 states did not expand. S1 Table lists the states with Medicaid expansion status as of September 2018 [23].

Previous evidence gave mixed results regarding the impact of Medicaid expansion on utilization of mammography and Pap tests [24–29]. Expansion experiences from pre-ACA anticipated positive effect on screening rates [26–28], while post-ACA either did not find significant impact [24,25] or found a favorable results when analyzing utilization in a community health center [29]. In any case, these studies had a short follow-up time after the introduction of ACA which perhaps made it difficult to detect significant change. Also, the individuals’ lack of knowledge/awareness of preventive care benefits provided by the ACA [30] may explain the limited impact of the coverage expansion on their use of these services.

Since no conclusive evidence is available, this study made an attempt to understand the effect of Medicaid expansion on probability of obtaining screening tests like mammography and Pap tests among low-income women. Since the study is using nationally representative data set, the results would indicate the effects of policy change for the country as a whole. Nationally representative data will also allow identification of factor affecting utilization rates.

## Materials and methods

### Data source

Data for this study was obtained from the Medical Expenditure Panel Survey—Household Component MEPS-HC [31]. The MEPS is a set of large-scale surveys which collects data from a sample of families and individuals in selected communities across the United States, drawn from a nationally representative subsample of households. The MEPS contains the data on health care utilization, health insurance status, and coverage source that are required to answer the research question of the study. The combined average response rate for the years 2012–2016 was 50.7% [32]. The study was reviewed and approved by the Office of Research Compliance, an administrative office that supports the University of South Carolina Institutional Review Board (USC IRB). All data used was fully anonymized before it was accessed.

### Sample

From the 2012–16 MEPS datasets, the sample extracted consists of nonelderly low-income women living in the U.S. The sample size was 6,427 in expansion states (3,459 pre-ACA and 2,968 post-ACA) and 6,831 in non-expansion states (3,729 pre-ACA and 3,102 post-ACA). Women living in states that already provided Medicaid or similar coverage to low-income adults before ACA’s Medicaid expansion in 2014 were excluded from the analysis (District of Columbia, Delaware, Massachusetts, New York, and Vermont). Women aged 65 years or older were excluded because they are eligible for Medicare. Women belonging to low-income households, as defined by the ACA, were selected for the analysis as this group is eligible for participation in Medicaid after the policy change, if they were not enrolled in Medicaid at the time of expansion. In accordance with screening guidelines, the mammography cohort will include women aged 40–64 and the Pap test cohort will include women aged 21–64. Women with concurrent or past diagnoses with breast or cervical cancer were excluded from the analysis to focus on utilization of screening services for preventive or early diagnosis purposes.

### Design

This study used a difference-in-difference (DID) design in a regression framework. This analytic design tests a comparison of the change in trends of outcomes before and after Medicaid expansion across expansion states vs non-expansion states, controlling for other covariates representing risk attitudes and preference structure. The treatment group includes women living in Medicaid expansion states and control group includes women living in non-expansion states. Only the states that expanded Medicaid between January 2014 and January 2016 were included in the treatment group (S1 Table). The states that already provided Medicaid or similar coverage to low-income adults before 2014 were excluded (District of Columbia, Delaware, Massachusetts, New York, and Vermont) (S1 Table). For estimating the DID parameters, pre-ACA period is defined as the years 2012–13 and the post period is defined as the years 2015–16.

The following multivariate linear regression was estimated to find the effect of the policy change on the outcome variables, the likelihood of receiving mammography and pap smears:

$$Y_{ist}=\beta_{0}+\beta_{1}Treatment+\beta_{2}Post+\beta_{3}(Treatment*Post)+\beta_{x}Covariates+\varepsilon$$

Where “Y_ist_” represents outcome for individual “i” living in state “s” at time ‘t”. β_0_ is the baseline average. The term “Treatment” is a dummy variable equal to 1 if the individual resides in a treatment group (expansion state). β_1_ is the difference between the two groups pre-intervention. The term “Post” is a dummy variable equal to 1 if the time is after the Medicaid expansion. β_2_ is the time trend in control group. The term “Treatment*Post” is an interaction term of intervention and time, β_3_ represents the difference-in-differences estimator capturing the effect of Medicaid expansion. Covariates are added to the model to control for preference structure and risk attitudes.

This regression model, in theory, will be able to indicate the effect of treatment if the intervention and control groups are identical at the baseline or show similar pattern of change over the years. In the real world, the intervention and control groups in pre-intervention period are never identical and therefore differences between the groups need to be explicitly considered and incorporated in the analysis. The effect of program change can be estimated if the assumption of similar pattern of changes over the years in pre-intervention years may be assumed in post-intervention periods as well. This is known as the “parallel assumption” in DID analysis. Since the parallel assumption must hold for an unbiased DID estimator, we can test the parallel movements or trend in the outcomes prior to policy change in treatment and control groups over a number of years. To assess the validity of this assumption, we regressed each outcome for the years 2005 to 2013 on variables indicating years, state expansion status and an interaction term of year and state expansion status. If the coefficient of the interaction term is not statistically different from zero, it implies that the rate of change of the dependent variables is not different between the intervention and control areas confirming the parallel movement of the outcome over the years prior to the implementation of the intervention.

### Outcome

The outcomes for this study are the self-reported receipt of mammogram, Pap test, and Medicaid enrollment status. For the preventive services, respondents were asked “About how long has it been since you had this mammogram/Pap test?” with possible responses being “within past year,” “within past 2 years,” etc. In accordance with screening guidelines, a dummy variable was created for mammogram utilization equal to 1 if the test was taken within 1 to 2 years, and a dummy variable for pap test utilization equals to 1 if the test was taken within 1 to 3 years.

### Covariates

We controlled for variables that we believe may modify the preference structure and risk attitude of women in the sample. According to the Demand Theory, demand for health services is a function of prices of the services, household income, preference structure, and risk-attitude. We chose covariates that may modify the preference structure and risk attitude, making individuals more risk averse and therefore more likely to undergo screening tests. The covariates chosen are: age, race, marital status, education, health insurance status, comorbidity, physical activity, smoking status, and metropolitan area.

### Statistical analysis

First, univariate analysis was done to produce baseline descriptive statistics of the low-income women living in treatment (expansion states) and control groups (non-expansion states). Second, we tested the parallel trends assumption across expansion and non-expansion states by regressing each outcome for the years 2005 to 2013 on variables indicating years, state expansion status and an interaction term of year and state expansion status. Third, a difference-in-differences regression model was estimated by linear ordinary least squares. A linear model was chosen to allow a direct interpretation of the coefficients and avoid interpretive issues inherent to interaction terms in nonlinear models [33,34]. The key parameter of interest from the DID model was the parameter associated with the interaction between treatment and time. This parameter represents the estimated difference in outcome rates between pre- and post-policy change, across states that were and were not affected by the policy change. Differences were considered statistically significant if P-value of t-statistics <0.05. Finally, a sub-group analyses was done using linear regression and univariate analysis to explain the effect of different demographics, socioeconomics, and geographic determinants on screening use. All analyses were carried out using STATA software version 14 (2015; Stata 14.0 Statistical Software, College Station, TX, USA). The analyses accounted for probability weighting in the MEPS [35,36] to obtain national estimates of effects of the policy change.

## Results

Table 1 shows the baseline characteristics of the nonelderly low-income women living in expansion and non-expansion states. Majority of the low-income women were white in both expansion and non-expansion states, however, more black women lived in non-expansion states (37.38%) compared to expansion states (23.94%). In both treatment and control groups, majority of low-income women did not have a college degree (Table 1). In expansion and non-expansion states, majority of low-income women had public health insurance, however, more women had public health insurance in expansion states (53.22%) as compared to non-expansion states (41.67%). Majority of the low-income women lived in metropolitan areas in both treatment and control groups (Table 1). Women in treatment and control groups had a similar average age (37). Therefore, states deciding to expand Medicaid were different from the states deciding not to expand in terms of percent of low income population not white, level of coverage of public insurance program and percent of poor women living in metro areas.

**Table 1:** Baseline characteristics of low-income women aged 18–64, living in expansion and non-expansion states, pre-ACA (2012–13), MEPS dataset.

Characteristic	Non-expansion states N = 3,729	Expansion states N = 3,459	P value
Age	37 (mean)	37 (mean)	0.0839
Race			0.000
White	57.39	67.07	
Black	37.38	23.94	
Other minorities	5.23	8.99	
Education			0.000
Some school	34.51	38.90	
High school	34.54	31.17	
College	30.95	29.94	
Health insurance			0.000
Private	18.10	16.48	
Public	41.67	53.22	
Uninsured	40.23	30.30	
Metropolitan area			0.000
Metro	82.38	87.80	
Non-metro	17.62	12.20	

**Fig 1:**
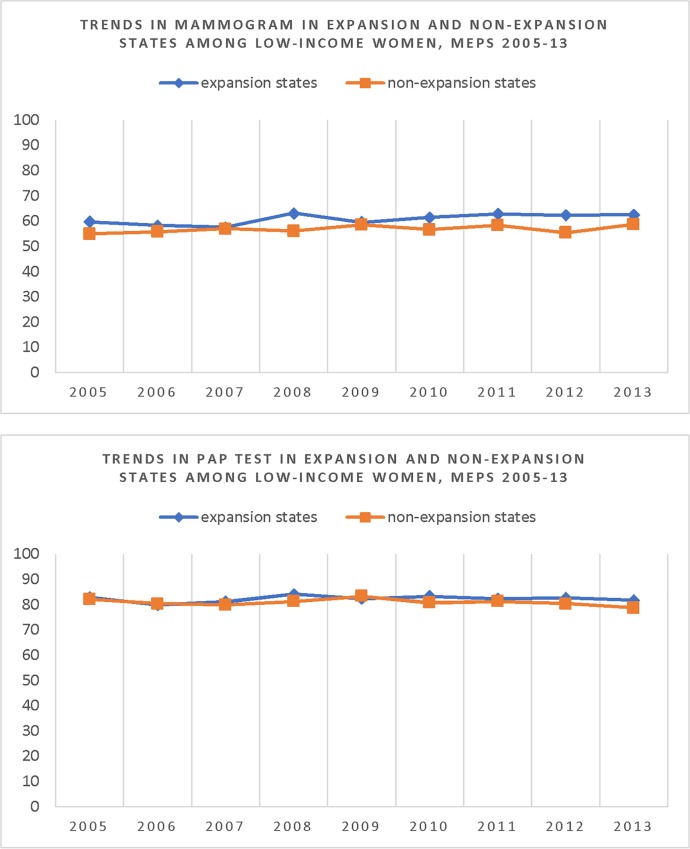
Trends in mammogram and Pap test uptakes in expansion and non-expansion states among low-income women, MEPS 2005–13.

Fig 1 shows trends in mammogram and Pap tests rates across expansion and non-expansion state for the years 2005 to 2013. Results from the regression that tested the parallel assumption of the time trend of outcome variable show that the slope of the trend functions were similar for these two groups of states prior to the implementation of the ACA policy on Medicaid expansion (S2 Table).

Results from the univariate analysis that examined screening rates among women living in expansion states post-ACA by different sub-groups can be found in S3 Table. The results show majority of low-income women who used mammograms and Pap tests were high-income (76.20%, 85.73%) high-education attainment (72.93%, 83.88%), Black (73.61%, 87.91%), with private insurance (74.20%, 84.13%), living in metropolitan areas (71.36%, 83.13%), and reported having a usual source of care (74.52%, 83.92%), for mammograms and Pap tests respectively.

Table 2 reports the results from the difference-in-difference adjusted regression model. The DID estimates indicate that the probability of enrolling to Medicaid has increased significantly among the low-income women after the implementation of Medicaid expansion (estimated coefficient 0.0889 with t-value 3.68). The DID estimates indicate that for the Medicaid expansion states the probability of utilizing mammograms did not change significantly (estimated coefficient -0.0476 with t-value -1.26) while for the Pap tests the probability of utilizing the test has decreased significantly among low-income women after the implementation of Medicaid expansion compared to non-expansion states (estimated coefficient -0.0615, t-value -2.76).

**Table 2:** Results from the difference-in-differences adjusted regression model, nonelderly low-income women (2012–16), MEPS dataset.

Outcome	Expansion states		Non-expansion states		Difference-in-differences
	Pre-ACA rate	Post-ACA rate	Pre-ACA rate	Post-ACA rate	
Mammogram	62.66%	64.69%	58.87%	61.77%	-0.0476 (-1.26)
Pap tests	81.90%	80.19%	78.80%	79.36%	-0.0615** (-2.76)
Medicaid enrollment	38.10%	52.31%	21.12%	25.03%	0.0889*** (3.68)

t statistics in parentheses

* p<0.05

** p<0.01

*** p<0.001

Table 3 shows the results on the likelihood of receiving mammograms and Pap tests among low-income women using a number of possible determinants of utilization of the screening tests. The sub-group analysis shows that poor women with higher age were more likely to receive mammograms (estimated coefficient 0.0102, t-value 4.16) and less likely to receive Pap tests (estimated coefficient -0.174, t-value -5.08). Black women were more likely to receive mammograms (estimated coefficient 0.0812, t-value 3.87) and Pap tests (estimated coefficient 0.0686, t-value 5.75) as compared to white women. The table also indicates that women from other minority population groups were less likely to receive Pap tests as compared to white women (estimated coefficient -0.0646, t-value -2.90). Women with a college degree were more likely to receive mammograms (estimated coefficient 0.0605, t-value 2.56) than those who had less than high school education. Women who were divorced were less likely to receive mammograms (coefficient -0.0875, t-value -3.47) and Pap tests (coefficient -0.0385, t-value -2.00) compared to married women. Women with public health insurance or no insurance (uninsured) were less likely than those with private insurance to receive mammograms (estimated coefficients -0.0654 and -0.250). No statistically significant difference was detected for Pap test use between women with public and private insurance (estimated coefficient -0.00391, t-value -0.26) although uninsured women were less likely to receive and Pap tests (estimated coefficient -0.110, t-value -6.61).

**Table 3:** Likelihoods of receiving mammograms and Pap tests using a number of determinants, results from adjusted linear regression model (2012–16), MEPS dataset.

Determinants	Mammogram	Pap test
Age (continuous)	0.0102*** (4.16)	0.000137 (0.11)
21–39	NA	Reference group
40–49	Reference group	-0.0789*** (-3.59)
50–64	-0.00818 (-0.23)	-0.174*** (-5.08)
Black(Reference group: White)	0.0812*** (3.87)	0.0686*** (5.75)
Other minorities(Reference group: White)	-0.0588 (-1.59)	-0.0646** (-2.90)
High school(Reference group: Some school)	0.0419 (1.82)	-0.00437 (-0.33)
College(Reference group: Some school)	0.0605* (2.56)	-0.0120 (-0.85)
Widowed(Reference group: Married)	-0.103* (-2.56)	-0.0883* (-2.47)
Divorced(Reference group: Married)	-0.0875*** (-3.47)	-0.0385* (-2.00)
Separated from spouse(Reference group: Married)	-0.0799* (-2.37)	-0.00593 (-0.27)
Never Married(Reference group: Married)	-0.0749** (-2.86)	-0.0277* (-2.03)
Public insurance(Reference group: Private insurance)	-0.0654* (-2.55)	-0.00391 (-0.26)
Uninsured(Reference group: Private insurance)	-0.250*** (-8.97)	-0.110*** (-6.61)
Non-metropolitan area(Reference group: Metropolitan area)	-0.0489 (-1.62)	-0.0615*** (-3.41)
Usual source of care not available(Reference group: Usual source of care not available)	-0.141*** (-4.03)	-0.0562** (-3.02)
Do not exercise regularly(Reference group: Exercise regularly)	-0.00171 (-0.09)	-0.0102 (-0.92)
Non-smoker(Reference group: Smoker)	0.0706** (3.15)	0.0446*** (3.30)
1 chronic disease(Reference group: No chronic disease)	0.0479 (1.68)	0.0223 (1.63)
+2 chronic diseases(Reference group: No chronic disease)	0.133*** (5.22)	0.0251 (1.58)

t statistics in parentheses

* p<0.05

** p<0.01

*** p<0.001

Women living in non-metropolitan areas were less likely than those in metropolitan areas to receive Pap tests (estimated coefficient -0.0615, t-value -3.41) but no difference was detected for mammogram use (estimated coefficient -0.0489, t-value -1.62). Women who reported not having a usual source of care were less likely to receive mammograms (estimated coefficient -0.141, t-value -4.03) and Pap tests (estimated coefficient -0.0562, t-value -3.02) compared to those who have a usual source of care. Non-smokers were more likely to receive mammograms (estimated coefficient 0.0706, t-value 3.15) and Pap tests (estimated coefficient 0.0446, t-value 3.30) as compared to smokers. Women with two or more chronic diseases were more likely to receive mammograms (estimated coefficient 0.133, t-value 5.22) but no differences were detected for Pap test use (estimated coefficient 0.0251, t-value 1.58).

## Discussion

The affordable care act (ACA) expanded Medicaid eligibility coverage to the entire low-income population in order to improve access and utilization among this disadvantage section of the population. In the years before the ACA, rates of mammograms and Pap tests showed declining trends among women and more so among poor women [11]. This study examined the impact of expanding health coverage through Medicaid on the rates of mammograms and Pap tests among poor women. The difference-in-difference (DID) estimates indicate that Medicaid enrollment has increased significantly among low-income women after the implementation of the Medicaid expansion (Table 2). This is a proximate measure of success of ACA in terms of providing coverage to poor women through Medicaid. Other studies also found increased Medicaid enrollment in expansion states compared to non-expansion states [37]. However, the increase in Medicaid enrollment among low-income women did not translate into increased rates of mammograms or Pap test utilization compared to poor women in non-expansion states. Other studies also found little impact of Medicaid expansion on mammography and Pap tests rates [37–39].

Although the difference-in-differences estimate did not show increase in mammograms and Pap tests rates, low-income women living in expansion states used more screenings than their counterparts in non-expansion states (Table 2). Historically, mortality rates of breast and cervical cancer were lower in the states that elected to expand Medicaid compared to those who elected not to expand [40]. We compared cancer burden in expansion vs non-expansion states in pre and post-ACA using data from the National Cancer Institute (NCI) and found that women in expansion states had lower mortality rates compared to women in non-expansion states (breast cancer: 20.13 vs 20.50; and cervical cancer: 1.97 vs 2.41) per 100,000 resident [40]. A previous study found that Southeastern states without Medicaid expansion tended to have higher cancer and lower screening rates and therefore disparities in cancer screening that already disfavor states with high cancer rates may widen in states that have chosen not to expand Medicaid [41].

A number of possible explanations can be advanced for this lack of improvements in mammograms and Pap tests rates among the low-income women despite gaining insurance coverage through Medicaid. First, poor knowledge/awareness about the availability of preventive benefits through ACA may result in low access to these services. There is evidence showing that newly insured individuals are not aware of the preventive services benefits of the ACA and thus do not use them [30]. Another study found that only 36.4% of adults reported knowing that the ACA requires coverage of certain preventive services without cost sharing [42]. Therefore, strategies aimed at improving public awareness about the availability of preventive services may be a vital tool in improving screening rates.

Second, in the U.S. health system, acute care takes priority over prevention. Studies have found that time constraints limit the ability of primary physicians to comply with preventive services recommendations [43]. A study has found that in states with higher Medicaid payments for office visits, Medicaid beneficiaries were more likely to be screened for breast and cervical cancer [44]. This indicate that increasing screenings among vulnerable population may be achieved through enhanced Medicaid reimbursements for physician consultations.

Third, our analysis show that low-income women with private insurance were more likely to receive mammograms than those with public insurance (Table 3). Also, in expansion states, the proportion of low-income women with private insurance used more mammograms and Pap tests than those with public insurance (S3 Table). Another study found that women with employer-based insurance/Medicare were more likely to get breast and cervical cancer screenings [45]. According to a survey conducted by the National Center for Health Statistics, only two out of three primary care physicians surveyed in 2011 were willing to accept new Medicaid patients [46]. Our analysis showed that women who reported having a usual source of care were more likely to receive mammograms and Pap tests than those without a usual source of care (Table 3). In expansion states, the proportion of low-income women who have a usual source of care used more mammograms and Pap tests than their counterparts in non-expansion states (S3 Table). Therefore, insurance-type and having a usual source of care appear to be more important in improving access to care and receiving these screenings.

A number of studies did find that Medicaid expansion was effective in improving utilization rates of certain preventive services such as glucose testing, cholesterol testing, and annual check-up, but not for cancer screenings [24,47]. The U.S. Preventive Services Task Force (USPSTF) guidelines for mammograms and Pap tests were updated around the time ACA was being implemented. The guidelines for mammograms were updated in 2009 to recommend mammograms for women aged 50–75 years every 2 years from the previous guidelines that recommended screening every 1–2 years for women aged 40 or older [48]. The guidelines for cervical cancer screenings was updated in 2012 to recommend the Pap test for women aged 21–65 every three years from the previous guidelines that recommended screening annually for women who are sexually active. This may explain the overall decline in cancer screenings in recent years. However, the effect of revised guidelines should be universal applicable to both expansion and non-expansion states and therefore may not explain the lack of effect on mammograms and Pap tests in expansion states in our difference-in-difference design.

It is also possible that the results from the difference-in-difference model are biased or washed-out because of the possibility that a significant number of poor women received screenings through other national programs such as the National Breast and Cervical Cancer Early Detection Program (NBCCEDP) through the Centers for Disease Control and Prevention (CDC). The program was established in 1990 to provide free and/or reduced cost mammograms and Pap tests to women with limited incomes and those who lack health insurance. Although number of women receiving those services through the NBCCEDP has decreased in 2015–16 compared to 2013 [49], low-income women still benefit from this program which may have negated the impact of ACA on mammograms and Pap tests rates in our difference-differences analysis. Between 2012 and 2017 the NBCCEDP program provided 740,108 Pap tests and 902,751 mammograms to low-income women [49]. During 2012 to 2017, this program provided 441,206 Pap tests (452 screenings per 100,000 woman) and 498,659 mammograms (511 screenings per 100,000 woman) in expansion states. During this same period, 398 and 573 per 100,000 woman received Pap tests and mammography respectively in non-expansion states through this program [49]. Mammograms provided to women in non-expansion states was about 12% higher than the rate in expansion states and the opposite is true for Pap tests (12% lower in non-expansion states). Such differences in coverage rates of an external program can potentially bias the estimation of the effect of ACA on mammograms and Pap tests uptakes.

Our empirical analysis showed that screenings occurred at much higher rates among the high-income high-educated women while the low-income women had the lowest utilization rates (S3 Table). Before the ACA, a study found that the low-income women were less likely to receive possibly lifesaving recommended cancer screenings [50]. A post-ACA study examined the impact of Medicaid expansion on disparities in cancer screenings and found that large gaps remain in access, particularly for low-income adults [51]. Our analysis showed that disparities in terms of using mammograms and Pap tests remained and may have actually become worse. This possibly implies that other factors beyond insurance coverage (in this case, provided through Medicaid) should be examined in order to better understand the reasons for the persistence of socioeconomic disparities.

Finally, our sub-group analyses helped shed light on the likelihood of receiving mammograms and Pap tests among low-income women using a number of possible determinants of utilization. As women get older they are more likely to receive mammograms and less likely to receive pap tests (Table 3). This is expected as evidence suggests that the benefits from mammograms are more evident for older women while benefits from Pap tests are more evident for younger women [52,53]. Low-income women living in metropolitan areas were more likely to receive mammograms and Pap tests in both expansion and non-expansion states (Table 3). This is expected as access to care is better in metropolitan areas than in non-metro areas. Black women were more likely than white women to receive a Pap test (Table 3) and in expansion states, the proportion of black women receiving mammograms and Pap tests were higher than white women (S3 Table). African American women in the U.S. are more likely to be diagnosed and die from breast and cervical cancer than white women, which may explain the increased use of the screenings [54].

### Limitations

We acknowledge some important limitations of this study. First, information about outcomes relied on self-reported survey responses which might be subject to recall error. However, the MEPS follow up with health providers to reduce the reporting bias but some errors may still remain, especially for procedures and tests requiring longer recall time frame. Second, the data used in the analysis are cross-sectional and comparison of cross-sectional data at different years is not same as observing changes in the outcomes for a cohort with the implementation of ACA. The study design made an attempt to tease-out the effect of policy changes through DID and in most cases DID approach can identify the effect of policy change even when the starting characteristics of the control and intervention groups are significantly different. Third, this study examined the initial 3-year period after the ACA Medicaid expansion provision and a longer time frame may be needed to be able to see the effects of policy changes on outcomes. Finally, there were changes in the USPSTF guidelines for breast cancer and cervical screening that occurred around the same time as the ACA provisions but it should not affect the results of DID. One of the important sources of bias that could not be corrected for in the empirical analysis is the provision of these screenings to poor women by a national program free of charge. If this national program in post-policy change years provided more emphasis on offering screenings in non-expansion states, it can potentially offset any positive effects of Medicaid expansion in DID modeling. In any case, this lack of relative improvements in cancer screenings in the Medicaid expansion states (compared to non-expansion states) is perplexing and would require supplementing the national data with the effects of alternative programs and other structural differences between these two groups of states.

## Conclusion

Our study shows that expansion of Medicaid under the ACA was associated with increased Medicaid enrollment but did not yield near-term improvements in the use of mammography and Pap tests among low-income women. Although the difference-in-differences did not show improvements in mammograms and Pap tests due to Medicaid expansion under ACA, low-income women living in expansion states used higher level of screenings than their counterparts in non-expansion states. Since Medicaid expansion did not affect these screening tests, policy makers need to examine other factors that may act as barriers in improving access and utilization. Some possible explanations for this lack of impact of the Medicaid expansion on mammograms and Pap tests are presented in the discussion section but we have no concrete evidence to conclusively say which factors have affected access to screening tests adversely in the expansion states compared to non-expansion states. It is also possible that a longer timeframe will be needed for a change to be manifested itself rather than the three-year time frame used here. Future research on provider availability and characteristics, insurance types, and geographical variations is warranted for a better understanding of the use of cancer screening procedures by the poor women in the USA.

## Supporting information

S1 Table State Medicaid expansion status as of September 2018 with the start date of expansion in the states (Source: Kaiser foundation website). (DOCX)

S2 Table Test of parallel trend assumption in expansion and non-expansion states prior to introduction of ACA Medicaid expansion. Interaction term of year and state expansion status.
(DOCX)

S3 Table Rates of mammograms and Pap tests use and change in the rates in expansion states in post-ACA years by individual characteristics: Results from univariate analysis (2012–16) of MEPS data. (DOCX)
