# Supplementary material for: Impact of Medicaid coverage expansion under the Affordable Care Act on mammography and pap tests utilization among low-income women
Source: PLoS One. 2019 Apr 3;14(4):e0214886. doi: 10.1371/journal.pone.0214886 (PMC6447234; doi:10.1371/journal.pone.0214886)
Supplement: S1 Table — (DOCX) [file pone.0214886.s001.docx]

**S1 Table. State Medicaid expansion status as of September 2018 with the start date of expansion in the states (Source: Kaiser foundation website)**

| **Medicaid Expansion Status** | **States** | **Start date** | **Income eligible level** |
| --- | --- | --- | --- |
| Adapted | Arizona, Arkansas, California, Colorado, Connecticut, Hawaii, Illinois, Iowa, Kentucky, Maryland, Minnesota, Nevada, New Jersey, New Mexico, North Dakota, Ohio, Oregon, Rhode Island, Washington, West Virginia | Jan 2014 | 138% |
|  | Michigan | Apr 2014 | 138% |
|  | New Hampshire | Aug 2014 | 138% |
|  | Pennsylvania | Jan 2015 | 138% |
|  | Indiana | Feb 2015 | 138% |
|  | Alaska | Sep 2015 | 138% |
|  | Montana | Jan 2016 | 138% |
|  | Louisiana | Jul 2016 | 138% |
|  | Virginia | Jan 2019 | 138% |
|  | Maine | TBD | 138% |
| Did not adapt | Alabama, Florida, Georgia, Kansas, Mississippi,  Missouri, North Carolina, Oklahoma, South Carolina, South  Dakota, Tennessee, Texas, Wisconsin, Wyoming | NA | NA |
| Considering | Idaho, Nebraska, Utah | TBD | 138% |
| Adapted before 2014 | District of Columbia | Jul 2010 | 215% |
|  | Vermont | Jan 2011 | 160% |
|  | New York | Apr 2009 | 100% |
|  | Delaware | Jan 2009 | 100% |
|  | Massachusetts | Apr 2009 | 133% |
