# Supplementary material for: Impact of Medicaid coverage expansion under the Affordable Care Act on mammography and pap tests utilization among low-income women
Source: PLoS One. 2019 Apr 3;14(4):e0214886. doi: 10.1371/journal.pone.0214886 (PMC6447234; doi:10.1371/journal.pone.0214886)
Supplement: S2 Table — Interaction term of year and state expansion status. (DOCX) [file pone.0214886.s002.docx]

**S2 Table. Test of Parallel Trend Assumption in expansion and non-expansion states prior to introduction of ACA Medicaid Expansion**

| Outcome | Coef.  Interaction variable ^a^ | Std. Err | t | P>\|t\| | [95% conf. Interval] | |
| --- | --- | --- | --- | --- | --- | --- |
| Mammogram | .0039422 | .0188091 | 0.21 | 0.834 | -.0329256 | .04081 |
| Pap test | -.0163429 | .0111188 | -1.47 | 0.142 | -.0381361 | .0054502 |

^a^ Interaction term of year and state expansion status.
