# Supplementary material for: Impact of Medicaid coverage expansion under the Affordable Care Act on mammography and pap tests utilization among low-income women
Source: PLoS One. 2019 Apr 3;14(4):e0214886. doi: 10.1371/journal.pone.0214886 (PMC6447234; doi:10.1371/journal.pone.0214886)
Supplement: S3 Table — (DOCX) [file pone.0214886.s003.docx]

**S3 Table. Rates of mammograms and Pap tests use and change in the rates in expansion states in post-ACA years by individual characteristics: results from univariate analysis (2012-16) of MEPS data**

| Individual characteristics | Mammogram | | Pap test | |
| --- | --- | --- | --- | --- |
|  | % in post-ACA | Change 2012 to 2016 in % | % in post-ACA | Change 2012 to 2016 in % |
| Income: Low-income | 64.69 | + 2.03 | 80.19 | -1.71 |
| Middle-income | 70.08 | - 3.14 | 81.05 | -0.71 |
| High-income | 76.20 | -3.1 | 85.73 | -1.16 |
| Race: White | 70.10 | -0.99 | 81.49 | -0.97 |
| Black | 73.61 | -1.34 | 87.91 | -.04 |
| Other | 69.02 | +1.22 | 79.77 | -2.36 |
| Education: Some school | 65.23 | +0.20 | 80.93 | -0.56 |
| High school | 69.24 | +1.32 | 79.51 | +0.33 |
| College | 72.93 | -2.31 | 83.88 | -1.7 |
| Area: Metro | 71.36 | -0.87 | 83.13 | -0.96 |
| Non-metro/rural | 61.42 | -2.12 | 73.33 | -3.14 |
| Insurance: Private | 74.20 | -3.49 | 84.13 | -1.99 |
| Public insurance | 67.10 | -4.03 | 80.86 | -4.9 |
| Uninsured | 52.87 | +2.04 | 73.72 | +0.93 |
| Have usual source of care | 74.52 | -1.91 | 83.92 | -2.08 |
| Do not have usual source of care | 48.96 | +0.83 | 75.64 | +0.47 |
